# Study on the correlation between urinary retinol-binding protein and nonalcoholic fatty liver disease

**DOI:** 10.5937/jomb0-24666

**Published:** 2021-01-26

**Authors:** Chuang Li, Weiwei Kong, Lixia Kang, Tiehan Zhang, Weiqun Zhang, Weidong Wang

**Affiliations:** 1 Xinxiang Medical University, The Third Affiliated Hospital, Department of Laboratory Medicine, Xinxiang, Henan, China

**Keywords:** nonalcoholic fatty liver disease, renal injury, retinol-binding protein, retinol-vezujući protein, renalne povrede, nealkoholna masna jetra

## Abstract

**Background:**

Nonalcoholic fatty liver disease (NAFLD) affects human health worldwide. Our objective was to explore the correlation between urinary retinol-binding protein (URBP) and NAFLD.

**Methods:**

This cross-sectional study included 445 NAFLD patients and 911 healthy controls. The URBP level and other parameters were measured.

**Results:**

The URBP level (expressed by the RBP/creatinine ratio) was higher in the NAFLD patients compared with the non-NAFLD patients. The urinary RBP/creatinine ratio was an independent risk factor for NAFLD after univariate and multivariate regression analysis, with the or values of 2.271 (1.795-2.872, P < 0.001) and 2.338 (1.775-3.080, P < 0.001), respectively. The prevalence of the urinary RBP/creatinine ratio (groups 1, 2, 3, 4) was 20.0%, 17.3%, 27.3%, and 35.4%, respectively (P < 0.001), and the prevalence of NAFLD in the high urinary RBP/creatinine ratio group was significantly higher than that in the low urinary RBP/creatinine ratio group.

**Conclusions:**

Our results revealed that the urinary RBP/creatinine ratio was an independent risk factor for NAFLD.

## Abstract (sr)

**Uvod:**

Nealkoholna masna jetra (NAFLD) ima negativan uticaj na zdravlje ljudi širom sveta. Naš rad je bio usmeren na istraživanje povezanosti između urinarnog retinol-vezujućeg proteina (URBP) i nealkoholne masne jetre.

**Metode:**

Istraživanje, sprovedeno kao studija preseka (cross-sectional study), obuhvatilo je 445 pacijenata sa dijagnozom nealkoholne masne jetre i 911 zdravih osoba u kontrolnoj grupi. U istraživanju su mereni nivo urinarnog retinol-vezujućeg proteina (URBP) kao i drugi parametri.

**Rezultati:**

Nivo urinarnog retinol-vezujućeg proteina (koji se izražava kao odnos retinol-vezujućeg proteina i kreatinina) bio je viši kod pacijenata sa dijagnozom nealkoholne masne jetre nego kod onih bez ove dijagnoze. Odnos urinarnog retinol-vezujućeg proteina i kreatinina pokazao se kao nezavisan faktor rizika za nealkoholnu masnu jetru, nakon regresionih analiza sa jednom i više varijabli, sa dobijenim vrednostima koeficijenta korelacije (OR) koje redom iznose - 2,271 (1,795-2.872, P < 0,001) i 2,338 (1,775-3,080, P < 0,001). Učestalost odnosa urinarnog retinol-vezujućeg proteina i kreatinina (u grupama 1, 2, 3 i 4) bila je, redom, 20,0%, 17,3%, 27,3% i 35,4% (P < 0,001), a učestalost nealkoholne masne jetre u grupi sa visokim od no som bila je znatno veća nego u grupi sa niskim odnosom.

**Zaključak:**

Rezultati istraživanja otkrili su da je odnos urinarnog retinol-vezujućeg proteina i kreatinina nezavisan faktor rizika za nealkoholnu masnu jetru.

## Introduction

Nonalcoholic fatty liver disease (NAFLD) refers to a clinicopathologic syndrome which is characterized by the excessive deposition of intrahepatic fat, except for that caused by alcohol and other welldefined liver damage factors. NAFLD includes a series of interrelated pathological changes ranging from simple fatty liver to nonalcoholic steatohepatitis, liver fibrosis, and cirrhosis [1]. The disease is a clinically common chronic liver disease, and its occurrence and development are closely related to factors such as lipid metabolism disorders, increased proinflammatory factors, type 2 diabetes, and metabolic syndrome [2].

With the continuous improvement in people's living standards, the accompanying factors such as a diet structure, behaviour patterns, and consumption patterns have all changed substantially. The prevalence of some metabolic-related diseases has increased. NAFLD, a chronic metabolic disease, has become the most common liver disease in the world and has a serious impact on social health. The prevalence of NAFLD has reached 25-30% in Western countries [3] [4]. The prevalence of NAFLD in China is about 20% [5]. Most cases of simple steatosis do not evolve into nonalcoholic steatohepatitis, and only nonalcoholic steatohepatitis has a higher risk of developing into cirrhosis or hepatocellular carcinoma, and it has the most complications [6]. Therefore, predicting the development of NAFLD at an early stage will help to control its development. Current research shows that alanine aminotransferase (ALT), aspartate aminotransferase (AST) and γ-glutamyltransferase (GGT) are closely related to the development of NAFLD [7] [8]. Retinol-binding protein (RBP) is a low molecular weight liver-synthesized protein, and whose unbound form is freely filtered at the kidney glomerular level [9]. Recently, researchers have also found that retinol-binding protein 4 (RBP4) is closely related to the occurrence and development of NAFLD [10]. So far, there are few studies on the association between URBP (expressed by RBP/creatinine ratio) and NAFLD. The goal of this study was to investigate the relationship between URBP and NAFLD.

## Materials and Methods

### Subjects

The participants enrolled in the study were from the Physical Examination Center of First Affiliated Hospital of Zhejiang University in Hangzhou. From May to June 2016, the study initially included 1568 patients. After abdominal ultrasonography, 552 patients with NAFLD and 1016 patients with non-NAFLD were left. The diagnosis of NAFLD was made according to the guideline presented by the Fatty Liver Disease Study Group of the Chinese Liver Disease Association [11]. Patients were excluded for the following reasons: (i) alcohol consumption, > 140 g/week for men and > 70 g/week for women; (ii) the history of viral hepatitis, autoimmune hepatitis, or other forms of chronic liver disease; (iii) acute or chronic infections; (iv) existing systemic diseases that may cause fatty liver; (v) those with a history of kidney dysfunction or eGFR < 90 mL/min/1.73 m^2^. The remaining 1,356 eligible subjects (445 NAFLD and 911 non-NAFLD patients) were included in the study.

### Clinical and Anthropometric Parameters

Medical history and healthy habits were recorded by professional doctors. Weight, height, systolic blood pressure (SBP) and diastolic blood pressure (DBP) were measured, and body mass index (BMI) was calculated as weight in kilograms divided by height in meters squared. Experienced B-ultrasound doctors blinded to the clinical and laboratory data performed liver ultrasound examinations using an American ATL-HDI 5000 Color Doppler diasonograph and a 3.5 MHz B ultrasonic probe (Philips, New York, USA).

### Biochemical Analysis

Fasting blood samples, collected from all of the study subjects, were used both as a hematological index and for biochemical analysis. Mid-stream urine was obtained in the morning for the URBP measurements. Commercial assay kits (Chongqing Zhongyuan Biotechnology Limited Company, Chongqing, China) were used to measure RBP levels, and the detection method was latex particle-enhanced immune-turbidimetry. The normal level of URBP ranged from 0.00 to 0.70 mg/L. All samples were analyzed using Hitachi 7600 biochemical analyzer (Hitachi, Tokyo, Japan) and Sysmex XE-2100 automatic analyzer (Sysmex, Kobe, Japan) by professional laboratory medical personnel.

### Data and Statistical Analysis

Statistical analyses were performed using SPSS, version 26.0 (SPSS, Chicago, IL, USA). The normality of the continuous data was verified using the Kolmogorov-Smirnov test. Normally distributed data are presented as the mean ±SD or as the median (first quartile-third quartile) if the distribution was skewed. Differences between groups were analyzed using the Student's t-test or the Mann-Whitney U-test, while a χ^2^ test was used for comparisons of count data. Logistic regression analysis was used to evaluate the risk factors for NAFLD. P values < 0.05 were considered as statistical significance.

### Ethics Statement

This study was approved by the Hospital Ethics Committee and was performed in accordance with the Declaration of Helsinki. Informed consent was obtained from all the subjects before participation.

## Results

### The characteristics of the subjects

The demographic and laboratory characteristics of participants enrolled in the study are shown in Table 1. We found that patients with NAFLD had significant differences in terms of gender, age, SBP, DBP, BMI, white blood cell (WBC), hemoglobin (HGB). As expected, urinary RBP/creatinine ratio in NAFLD patients was higher than that in the control group (median, 0.32 mg/g vs 0.21 mg/g, P < 0.001).

**Table 1:** Participant baseline characteristics NAFLD, nonalcoholic fatty liver disease; SBP, systolic blood pressure; DBP, diastolic blood pressure; BMI, body mass index; ALT, alanine aminotransferase; AST, aspartate aminotransferase; GGT, gamma-glutamyltransferase; WBC, white blood cell; HGB, hemoglobin; TG, triglyceride; Tch, total cholesterol; HDL-C, high-density lipoprotein cholesterol; RBP, retinol-binding protein; M, male; F, female

Variable	Subjects with NAFLD (n = 445)	Controls (n = 911)	P-value
Gender (M/F)	347/98	513/398	< 0.001
Age (y)	53 (45–62)	46 (39–58)	< 0.001
SBP (mmHg)	131 (120–142)	121 (110–132)	< 0.001
DBP (mmHg)	81 (74–87)	74 (66–82)	< 0.001
BMI (kg/m^2^)	26.11 (24.43–28.27)	22.28 (20.56–24.08)	< 0.001
ALT (U/L)	18 (12–27)	18 (13–25)	0.312
AST (U/L)	20 (17–24)	19 (17–24)	0.219
GGT (U/L)	20 (13–33)	20 (13–33)	0.950
WBC (10^9^/L)	5.7 (4.9–6.6)	5.3 (4.6–6.3)	< 0.001
HGB (g/L)	153 (142–162)	146 (134–158)	< 0.001
TG (mmol/L)	1.17 (0.80–1.84)	1.12 (0.78–1.68)	0.127
Tch (mmol/L)	4.55 (3.92–5.13)	4.55 (4.02–5.13)	0.470
HDL-C (mmol/L)	1.24 (1.05–1.51)	1.3 (1.08–1.54)	0.027
eGFR (mL/min/1.73 m^2^)	104.24 (97.79–113.68)	100.78 (95.62–107.98)	< 0.001
Urinary RBP/creatinine ratio (mg/g)	0.32 (0.16–0.67)	0.21 (0.12–0.39)	< 0.001

### The association between the urinary RBP/creatinine ratio and NAFLD

We used a logistic regression analysis to explore the risk factors for NAFLD. The results of the univari-ate regression analysis showed that gender, SBP, DBP, BMI, WBC, HGB and URBP were all risk factors for NAFLD (P < 0.05; Table 2). We put gender, age, SBP, DBP, BMI, ALT, AST, GGT, WBC, HGB, high-density lipoprotein cholesterol (HDL-C), triglyceride (TG), total cholesterol (Tch) and URBP into the multivariate logistic regression analysis. Table 3 shows the results of the adjusted multivariate logistic regression analysis models. These data show that urinary RBP/creatinine ratio was an independent risk factor for NAFLD prediction (2.338 (1.775-3.080), P < 0.001).

**Table 2:** Odds values (95% CI) of univariate regression analysis for predicting risk factors in NAFLD participants OR, Odds ratio; 95%CI, 95% confidence interval; NAFLD, nonalcoholic fatty liver disease; RBP, retinol-binding protein

Variable	Univariate (95% CI)	P-value
Gender	2.747 (2.119–3.561)	< 0.001
SBP	1.035 (1.027–1.042)	< 0.001
DBP	1.054 (1.043–1.065)	< 0.001
BMI	1.722 (1.619–1.832)	< 0.001
ALT	1.004 (0.998–1.011)	0.204
AST	1.006 (0.998–1.020)	0.114
GGT	0.999 (0.995–1.002)	0.479
WBC	1.251 (1.146–1.367)	< 0.001
HGB	1.024 (1.116–1.032)	< 0.001
TG	1.020 (0.926–1.124)	0.684
Tch	0.967 (0.847–1.104)	0.619
HDL-C	0.728 (0.517–1.025)	0.069
Urinary RBP / creatinine ratio	2.271 (1.795–2.872)	< 0.001

**Table 3:** Multivariate logistic regression was used to analyze the predictive value of urinary RBP/creatinine ratio for NAFLD OR, Odds ratio; 95%CI, 95% confidence interval; NAFLD, nonalcoholic fatty liver disease; RBP, retinol-binding protein

Model	OR (95%CI)	P-value
Gender, age, SBP, DBP, BMI	2.302 (1.749–3.030)	< 0.001
Adjusted for gender, age, SBP, DBP, BMI, ALT, AST	2.311 (1.757–3.041)	< 0.001
Adjusted for gender, age, SBP, DBP, BMI, ALT, AST, GGT, WBC	2.331 (1.766–3.078)	< 0.001
Adjusted for gender, age, SBP, DBP, BMI, ALT, AST, GGT, WBC, HGB, TG	2.324 (1.765–3.059)	< 0.001
Adjusted for gender, age, SBP, DBP, BMI, ALT, AST, GGT, WBC, HGB, TG, Tch and HDL-C	2.338 (1.775–3.080)	< 0.001

### The prevalence of NAFLD at different urinary RBP/creatinine ratio levels

To investigate the association of urinary RBP/creatinine ratio with NAFLD, we stratified subjects into quartiles according to their urinary RBP/creatinine ratio levels (< 0.13 mg/g, 0.14-0.23 mg/g, 0.24-0.46 mg/g, > 0.46 mg/g). We calculated the prevalence of NAFLD in the four groups (1, 2, 3, and 4). The prevalence related to NAFLD in the four groups was 20.0%, 17.3%, 27.3%, and 35.4%, respectively (tendency P <0.001; OPREZ Slika 1). This result indicates that the prevalence of NAFLD is significantly associated with the urinary RBP/creatinine ratio level, and the probability of developing NAFLD increases with the increase of the urinary RBP/creatinine ratio level.

**Figure 1:**
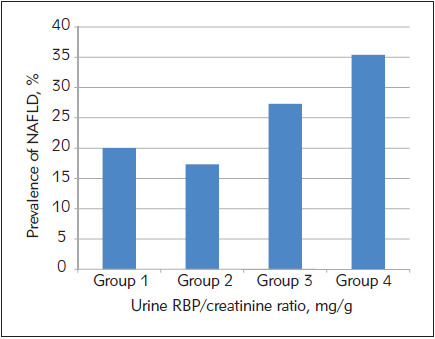
The prevalence of NAFLD at different urine RBP/creatinine ratio levels

## Discussion

To date, there have been relatively few studies on the relationship between urinary RBP/creatinine ratio and NAFLD. The present study showed that the urinary RBP/creatinine ratio level was significantly correlated with NAFLD even with normal-range eGFR. Urinary RBP/creatinine ratio was an independent risk factor for NAFLD by univariate and multiple logistic regression analyses. When the urinary RBP/creatinine ratio level was > 0.46 mg/g, the prevalence of NAFLD increased significantly, reaching 35.4%. This indicates that the incidence of NAFLD increases as the urinary RBP/creatinine ratio levels increase.

At present, the mechanism of interaction between urinary RBP/creatinine ratio and NAFLD is not well understood, but some possible associations have been proposed. It is generally believed that the »second strike theory« is the main pathogenesis of NAFLD. The first attack results from lipid accumulation caused by insulin resistance (IR), which causes fatty degeneration [12]. The second strike is the regulation of liver damage repair caused by steatosis. Regulatory processes such as oxidative stress, mitochondrial dysfunction, and inflammatory response further lead to fatty hepatitis, liver fibrosis, and related complications [13]. Retinol-binding protein (RBP) is a low molecular weight protein (21 kDa), and it is the only specific transporter of vitamin A in the blood [14]. Most RBP circulates in the plasma bound to transthyretin (TTR), and it cannot undergo glomerular filtration. Only 4-5% is free to undergo glomerular filtration, and retained and degraded by the proximal tubules [15]. Therefore, if a lot of RBP appears in the urine, it might mean kidney damage. Recently, there has also been ample evidence that there is a potential link between NAFLD and renal function [16]. Firstly, some data suggest that NAFLD may be the source of pro-inflammatory factors, including fetuin-A, plasminogen activator inhibitor-1 (PAI-1) and human fibroblast growth factor-21 (FGF-21), which may cause kidney damage [17] [18] [19]. Secondly, NAFLD may damage the kidneys by excessive secretion of very-low-density lipoprotein (VLDL) [20]. In addition, we all know that IR is a causative factor in NAFLD [21], and excessive IR activates the sympathetic nervous system and the renal angiotensin system, causing kidney damage [22]. Interestingly, Retinol-binding protein 4 (RBP4) can cause IR by stimulating the production of pro-inflammatory cytokines and/or interfering with normal insulin signalling [23], which is an important risk factor for the pathogenesis of NAFLD [24]. This might explain why RBP can increase in the urine of NAFLD and aggravate the progression of NAFLD.

It is well known that NAFLD jeopardizes the health of humans worldwide [25]. If the disease is diagnosed and treated in early stages, it will have a positive impact on the patient's prognosis and economy. Our subjects are NAFLD patients with normal eGFR, which means that the urinary RBP/creatinine ratio may increase when NAFLD causes mild kidney damage. Our results showed that the urinary RBP/creatinine ratio level was significantly associated with the prevalence of NAFLD, and the urinary RBP/creatinine ratio was an independent risk factor for NAFLD. Therefore, our data provide evidence for the urinary RBP/creatinine ratio to be regarded as a detectable urine marker for early kidney damage caused by NAFLD. Yanhong Yuan et al. [26] reported that URBP is an independent risk factor for the prognosis in patients with acute-on-chronic renal injury, and can be used for reversible detection of acute-onchronic renal injury. We propose the urinary RBP/creatinine ratio as a reversible test for NAFLD treatment, which requires further study.

Currently, liver biopsy is the gold standard for the diagnosis of NAFLD [27], but liver biopsy has some adverse complications, so it is not suitable for large-scale epidemiological examination [28]. Although a large number of non-invasive methods for diagnosing NAFLD have been applied [29], such as ultrasound and some blood markers, the ultrasound diagnosis requires experts with extensive clinical experience, and the blood tests may also have risks such as cross-contamination. As a urine marker, the urinary RBP/creatinine ratio has the advantages of being an easily obtained, mature technology and easily accepted by patients. Obviously, urine markers have a broader development space.

Our research has some limitations. The diagnosis of NAFLD is based on ultrasound results. Although it is widely accepted for the diagnosis of NAFLD, ultrasound is not sensitive enough for mild steatosis. Ultrasound cannot replace pathological studies as the gold standard in disease diagnosis, but the literature reports that ultrasound diagnosis and liver biopsy have a good correlation [11]. Our report is a crosssectional study that does not determine the causal relationship between clinical urinary RBP/creatinine ratio levels and NAFLD. Further prospective studies are needed to demonstrate that the urinary RBP/creatinine ratio is an independent risk factor for NAFLD.

## Conclusions

Our study showed a positive correlation between the urinary RBP/creatinine ratio levels and NAFLD, and the elevated urinary RBP/creatinine ratio levels, which suggests a greater likelihood of NAFLD. Hepatitis is prevalent in China, and NAFLD is currently a popular research topic. High levels of the urinary RBP/creatinine ratio were shown to have important significance for the prevention and diagnosis of NAFLD.

## Acknowledgements

The authors wish to thank the Clinical Laboratory at the First Affiliated Hospital of Zhejiang University for providing training during this research.

## Financial Disclosure

The authors declared that this study has received no financial support.

## Conflict of interest statement

The authors declare that they have no conflicts of interest in this work.
